# Effects of Whole-Body Cryotherapy in Comparison with Other Physical Modalities Used with Kinesitherapy in Rheumatoid Arthritis

**DOI:** 10.1155/2015/409174

**Published:** 2015-10-21

**Authors:** Małgorzata Gizińska, Radosław Rutkowski, Wojciech Romanowski, Jacek Lewandowski, Anna Straburzyńska-Lupa

**Affiliations:** ^1^Department of Physiotherapy, University School of Physical Education in Poznań, Królowej Jadwigi 27/39, 61-871 Poznań, Poland; ^2^Rheumatological Centre in Śrem, Mickiewicza 95, 63-100 Śrem, Poland; ^3^Department of Locomotor System Rehabilitation, University School of Physical Education in Poznań, Królowej Jadwigi 27/39, 61-871 Poznań, Poland

## Abstract

Whole-body cryotherapy (WBC) has been frequently used to supplement the rehabilitation of patients with rheumatoid arthritis (RA). The aim of this study was to compare the effect of WBC and traditional rehabilitation (TR) on clinical parameters and systemic levels of IL-6, TNF-*α* in patients with RA. The study group comprised 25 patients who were subjected to WBC (−110°C) and 19 patients who underwent a traditional rehabilitation program. Some clinical variables and levels of interleukin-6 (IL-6) and tumor necrosis factor-*α* (TNF-*α*) were used to assess the outcomes. After therapy both groups exhibited similar improvement in pain, disease activity, fatigue, time of walking, and the number of steps over a distance of 50 m. Only significantly better results were observed in HAQ in TR group (*p* < 0.05). However, similar significant reduction in IL-6 and TNF-*α* level was observed. The results showed positive effects of a 2-week rehabilitation program for patients with RA regardless of the kind of the applied physical procedure.

## 1. Introduction

Rheumatoid arthritis (RA) is a chronic, autoimmune, systemic connective tissue disease whose etiology is not fully understood. RA is more frequently observed in women and elderly people. The disease is characterized by nonspecific inflammation of the symmetrical joints, the occurrence of extra-articular changes, and organ damage that leads to disability and premature death [1]. Cytokines with well-known proinflammatory effects, especially interleukin-6 (IL-6) and tumor necrosis factor-*α* (TNF-*α*), play key roles in local and systemic manifestations of RA [2, 3].

This disease, despite treatment, has a chronic course with periods of exacerbation and remission. The inflammatory process begins in the synovium of joints and progresses to joint destruction, deformation, disability, and early death [4]. The destruction of the joints is different for each patient, and it is impossible to predict its progress [5]. The consequences of ongoing RA are pain, impaired physical function, and fatigue, which cause limitations in physical functioning and work disabilities, and finally adversely affect the health-related quality of life [6].

Depending on the clinical form and dynamics of the disease, comprehensive treatment is used to reduce disease activity and pain, prevent joint damage and loss of joint function, and facilitate the preservation of the ability to work, to participate in recreational activity, and to have a satisfactory quality of life [7]. For these purposes, in addition to pharmacotherapy, exercise therapy [8, 9] and many physical agents are used [10]. The basis of these treatment programs is kinesitherapy adapted to the conditions of and opportunities for the patient. As an adjunct to kinesitherapy, many locally applied physical agents play an important role in the treatment of RA symptoms [11]. Many authors have reported that thermotherapy, ultrasound, and laser are the most effective treatments [12–14] as well as magnetotherapy [15]. Some reports have indicated that a properly matched physiotherapy intervention can be an effective means of improving the objective and subjective measures of inflammation and functional status in RA [16].

For many years, whole-body cryotherapy (WBC) has also been frequently used to supplement the rehabilitation of RA patients [17]. However, the effectiveness of this method remains controversial [18, 19]. Moreover, there are only a few studies in the literature comparing the effects of systemic and local cryotherapy [18, 20], and there is still lack of research comparing the effectiveness of WBC to other modalities.

The aim of this study was to investigate the effects of systemic cryotherapy in comparison with other modalities used with kinesitherapy on clinical parameters and systemic levels of IL-6, TNF-*α* in patients with RA.

## 2. Materials and Methods

The study group comprised 44 enrolled patients aged 55.8 ± 5.9 years. They were exclusively postmenopausal women who were admitted to the rheumatology department with the diagnosis of RA according to the American College of Rheumatology criteria [21], which were in force at the time of this research.

Patients with contraindications to physical treatments were excluded. This study was approved by the bioethics committee of the University of Medical Sciences in Poznan. All the participants provided informed consent.

The patients were subjected to comprehensive treatment that included pharmacotherapy, kinesitherapy, and physical modalities.

The patients were divided into two groups. The research group (group I), which received whole-body cryotherapy, consisted of 25 patients. Patients are wearing minimal clothing (a bathing suit), gloves, socks, shoes, and headband covering the ears. In addition, airway was secured with a surgical mask. They passed through prechambers (−10°C and −60°C) into the therapy-chamber (−110°C), where they stayed for 3 min, walking in a circles and performing energetic movements by the upper limbs.

Traditional rehabilitation group (group II) consisted of 19 patients who received a traditional rehabilitation program with other physical agents. The program included magnetotherapy, electrotherapy, ultrasound therapy, and laser therapy, which are recommended by international organizations against rheumatic diseases [22, 23]. Physiotherapy treatments were performed in accordance with generally applicable methods. The type of treatment and the dose were individually tailored for each patient.

The 2-week treatment program included procedures carried out once a day, every day, with a weekend break. A similar kinesitherapy program was used in both groups, which was individualized according to the patients' functional capabilities, overall health, age, and severity of disease. All exercises were carried out under the supervision of physical therapists.

The study was conducted twice, on the first and last days of stay in the rheumatology ward.

### 2.1. Pain and Fatigue

The visual analogue scale (VAS) was used to assess the pain severity and fatigue. The VAS results were obtained by measuring the distance in millimeters from the beginning of the scale to the position selected by the patient from 0 to 100 mm in witch 0 is “no pain or fatigue” and 100 is “the worst possible pain or fatigue” [24, 25].

### 2.2. Disease Activity Score 28

The disease activity score 28 (DAS28) included the number of swollen and tender joints, global VAS score assessed by the patient, and erythrocyte sedimentation rate [26].

### 2.3. Health Assessment Questionnaire-Disability Index

To examine physician function, the Health Assessment Questionnaire-Disability Index (HAQ-DI) was used. The HAQ-DI was the original HAQ developed and validated in the late 1970s. It evolved over numerous iterations through a series of subjective and objective assessments. The HAQ-DI is composed of 20 detailed questions about daily activities, divided into eight categories: dressing and taking care of appearance, arising, eating, walking, hygiene, reaching, gripping, and daily life activities. All respondents assessed their own difficulty in carrying out each activity on a scale from 0 to 3 (0 means no difficulty in performing the task and 3 means the task was impossible to perform) [27].

### 2.4. Fifty-Meter Walk Test

Each of the respondents performed a 50 m walk test. They were asked to begin walking at a normal (individual) speed at the start signal. After walking for 25 m in a straight line, they turned around and returned to the starting point. The time was recorded with a stopwatch (Sport Tester Polar RS 300X), and the number of steps taken over 50 m was counted [28].

### 2.5. Analytical Procedures

Fasting blood samples were taken from the antecubital vein and were centrifuged at 5,000 rpm at 4°C. The serum was separated and stored at −70°C. ESR was measured using Medlab Products kit (Poland); rheumatoid factor (RF) levels were detected using BioSystems kit (Spain). Serum concentrations of high-sensitivity TNF-*α* and high-sensitivity IL-6 were analyzed by immunoenzymatic ELISA (TNF-*α* and IL-6 assay kits, assay sensitivity = 0.038 pg/mL, 0.016 pg/mL, resp.; R&D Systems, UK) and were determined in case of 16 WBC and 14 TR group patients.

### 2.6. Statistical Analysis

The values are presented as means, standard deviations, medians, and interquartile ranges. To verify the hypothesis of a normal distribution of the analyzed variables, the Shapiro-Wilk test was used. Most of the variables significantly differed from a normal distribution. To further analyze variables with normal distribution, a parametric test (Student's *t*-test) and other nonparametric tests were used. To assess the significance of the differences between terms, the *t*-test or the Wilcoxon test was used. In addition, to determine the significance of differences between the two treatments, the *t*-test for independent groups and the Mann-Whitney *U* test were used. Finally, correlations between variables for all respondents were assessed by using the Spearman rank test.

The hypotheses were verified at the level of *p* < 0.05. Statistical analysis was performed by using the Statistica 8.0 package.

## 3. Results

The characteristics of the subjects by group are shown in Table 1.

The study groups did not differ from each other in terms of age, body mass index, DAS28 score, and duration of disease.

The clinical parameters after treatment with physiotherapy and the comparison of results between the two groups are presented in Table 2.

In both groups, after treatment, a significant reduction in the severity of pain (group I, *p* < 0.01; group II, *p* < 0.01), and in the duration of morning stiffness in the WBC group (*p* < 0.05), was observed.

In both groups after treatment, a significant reduction in the severity of fatigue (group I, *p* < 0.01; group II, *p* < 0.05) was also observed.

In addition, the DAS28 score was also significantly reduced in the two groups (group I, *p* < 0.01; group II, *p* < 0.05).

The HAQ-DI showed a significant reduction in specific difficulties in performing daily activities of life after treatment in groups I and II (*p* < 0.01), and it also showed significant differences between the two studied groups (group I versus group II, *p* < 0.05).

There was a statistically significant reduction in the time of walking (group I, *p* < 0.01; group II, *p* < 0.05) and number of steps (group I, *p* < 0.01; group II, *p* < 0.05) over a distance of 50 m.

There were no significant differences in effectiveness between the treatment groups except in the HAQ-DI.

In all patients, there were significant positive correlations between the severity of pain and duration of morning stiffness (*r* = 0.46; *p* = 0.0015), the severity of fatigue (*r* = 0.46; *p* = 0.0019), and DAS28 (*r* = 0.44, *p* = 0.0038). There was no significant correlation between fatigue and cytokines.

In both investigated groups there were significant, positive correlations between HAQ-DI and the severity of pain (*r* = 0.50, *p* = 0.0006), fatigue (*r* = 0.39, *p* = 0.0080), duration of morning stiffness (*r* = 0.37, *p* = 0.0136), and the DAS28 score (*r* = 0.50, *p* = 0.0007).

There were also correlations between HAQ-DI and time (*r* = 0.50, *p* = 0.0006) and number of steps (*r* = 0.51, *p* = 0.0004) and between fatigue and time (*r* = 0.30, *p* = 0.047) and number of steps (*r* = 0.33, *p* = 0.029).

Biochemical parameters in 2 groups of patients before and after the therapy are shown in Table 3.

In both groups after treatment, a significant reduction in level of IL-6 (WBC-group, *p* < 0.05; TR-group, *p* < 0.01) and TNF-*α* (group WBC, *p* < 0.05; group TR, *p* < 0.05) was observed. ESR was significantly reduced only in WBC group (group WBC, *p* < 0.05; group TR, n.s.).

## 4. Discussion

The results demonstrated that in two treatment groups, regardless of the physiotherapy treatment used, there were comparable significant improvements in the severity of pain, duration of morning stiffness, DAS28 score, fatigue, and walking time and the number of steps in the 50 m walking test. There was a significant improvement in functional status as assessed by using the HAQ; however, the improvement in the group subjected to the traditional model of physical procedures was significantly higher than in the group treated with cryotherapy. The results demonstrated that in both groups the levels of IL-6 and TNF-*α* decreased significantly.

One of the main symptoms of RA is pain, which, as highlighted by many authors, predominantly restricts all aspects of life [25, 29]. Our results showing a significant improvement in pain severity are consistent with the results of other authors who drew attention to the significant analgesic efficacy of physiotherapy treatments, including systemic cryotherapy [30, 31] and local [32, 33] treatments for RA. Also, Miller [20] and Hirvonen et al. [18] compared local and WBC treatments, and significantly better results in pain sensation were obtained in the group treated with systemic cryotherapy. Hirvonen et al. [18] point out that, despite the high efficiency and a small percentage of reported side effects, WBC treatment is the expensive and available only in properly prepared specialized centers. Adverse effects that may occur during therapy in cryogenic chamber include frostbite, headaches and dizziness, worsening pain, shortness of breath, and circulatory collapse [30]. However, none of these side effects were observed in our study. Miller [20] suggested that improvement in pain is related to the hormonal tuning of the body, increased secretion of endorphins, antidepressant action, and mobilization to undertake more physical activity by patients after their stay at low temperatures. Metzger et al. [31] concluded that the use of cryogenic temperature produces a considerable effect of pain reduction, which creates good conditions for exercise and occupational therapy. Although many other local physiotherapy treatments are commonly used in rheumatic diseases, few research on their therapeutic efficacy can be found. As shown by Segal et al. [15], magnetic therapy significantly reduces pain in the knee joints of patients with RA. De Dios Sancho and Martín-Nogueras [14] applied kinesitherapy, massage, phototherapy, thermotherapy, ultrasound therapy, and magnetic therapy and observed a reduction of pain by up to 50%. However, Falconer et al. [34] did not confirm a significant analgesic effect of ultrasound in their study, pointing rather to the efficacy of kinesitherapy in reducing pain. Research on the effectiveness of laser therapy in the treatment of pain did not yield clear results. In the study by Goats et al. [35] on low-level laser therapy, there was no difference in the severity of pain between the group treated with laser and the placebo group. This result could also be confirmed by studies showing that exercise already has an analgesic effect [36].

In our study, we did not find a significantly better analgesic effect of WBC compared with traditional physical therapy. This may suggest that comprehensive, monitored rehabilitation programs are important, regardless of the type of physical treatment. It should be noted that all physical treatments were tailored individually, taking into account the patients' condition, indications, contraindications, interindividual sensitivity, and treatment preference.

In patients with RA, morning stiffness is a common and important symptom that allows controlling the progression of the disease. After treatment, we observed a significant reduction in the duration of morning stiffness in the group treated with WBC, whereas only a trend in reduction was found in the group treated by using the traditional model. Comparison of the effectiveness of both treatments revealed no significant differences in morning stiffness. De Dios Sancho and Martín-Nogueras [14] observed a reduction in morning stiffness after various traditional physical treatments. In contrast, Goats et al. [35], in their study of the use of low-level laser therapy, did not find a significant change in morning stiffness in patients with RA.

DAS28 is a commonly used tool for the assessment of disease activity. In our study, after the treatment, a significant reduction in DAS28 score was observed in both groups, and there were no significant differences in the effectiveness of these therapies. Scientific reports are not clear in this context. Hirvonen et al. [18] observed no change in disease activity after 7 days of WBC treatment. In our study, the treatment procedure was longer (2 weeks), which could account for the positive results in terms of disease activity. Häkkinen et al. [30] showed that WBC results in a significant improvement in the DAS for only a short period after therapy; that is, there were no significant changes in DAS at 2 months after treatment. Still, little is known about the influence of other physical treatments on disease activity. Goats et al. [35] showed that laser therapy has no significant effect on the inflammatory markers characteristic of RA. Stojanović et al. [37] have shown that balneophysical treatment for RA has positive and therapeutic effects on disease activity, function, and health as measured by using the HAQ. It was observed that, after about 2 weeks of therapy, the DAS28 score dropped significantly. This seems to indicate the role of a complex procedure in RA. The question is whether kinesitherapy can affect the DAS28 score. Baillet et al. [38] pointed out that exercise can cause a positive effect but only if the heart rate increases to 60% during the first 20 min. In this study, significant effects were observed after one month of therapy, whereas at 6 and 12 months the changes were not significant. It should also be noted that, in our study, we did not evaluate exercise intensity, and therefore it was difficult to refer to the results of Baillet et al. [38].

One of the aims of therapy is to maintain the highest functionality possible in a patient. In the preliminary study, significant correlations between the HAQ-DI and pain, DAS28, duration of morning stiffness, fatigue severity, and duration of walk and number of steps over 50 m were reported in the whole group.

This confirms the observations of other authors who showed that the HAQ-DI reflects disease activity [28]. Häkkinen et al. [30] showed pain as the factor that is most strongly associated with disability, as examined by using an HAQ. In addition, Häkkinen et al. [39] showed a strong correlation between HAQ and the time of walking over a distance of 10 min in patients with RA.

After the treatments, the results of HAQ in both groups showed a significant reduction in the difficulty of performing various activities of daily living; significantly greater improvement was seen in patients subjected to the traditional model of rehabilitation. This appears to be associated with a greater degree of disability at baseline in this group, because greater therapeutic effects can be expected in subjects with more complaints and difficulties.

Physical measures of functional status, including the walking test, have been used in rheumatology clinical trials for a long time. The walking test measures the time and number of steps [28, 32] and can also monitor disease activity better than radiographic scores or laboratory tests [40, 41]. Häkkinen et al. [39] pointed out that a reduction in locomotion ability in patients with RA mainly affects pain, swelling, fatigue, joint structural changes, and gait disturbance. Our study shows significant correlations between the walking time and number of steps over a distance of 50 m, as well as between the walking time and number of steps and fatigue and HAQ. In our study, both groups demonstrated, after 2 weeks of therapy, a reduction in the walking time and number of steps over 50 m, and no differences were found between the groups.

Fatigue is a common factor that limits the overall function of patients with RA and reduces their quality of life [42]. Both fatigue and pain in the joints may be the result of estrogenic deprivation in postmenopausal women [43]. Other researchers have shown the relation of fatigue with disease activity, functional disability, general well-being, and mental state of patients [44, 45]. Our study confirmed, primarily, a significant correlation of perceived fatigue with the severity of disease, as reported by the patients on the DAS28 scale, and also with some objective parameters associated with functioning, such as time of walking and the number of steps during the 50 m march. In contrast, no significant correlation was found between fatigue and the level of IL-6. We have shown, regardless of the physical treatment used, a significant reduction in the perceived fatigue, which was comparable between both groups. Häkkinen et al. [30] draw special attention to the fact that WBC reduces fatigue and relieves pain and results in a subjective improvement in the patient, which are components of the healing process in this group of patients. Miller's [20] study compared WBC with local cryotherapy, showing that both treatments reduce pain but cryotherapy also reduces fatigue. However, Jastrząbek et al. [32] confirmed the positive impact on fatigue of a 10-day treatment with a variety of local cold treatments.

Effects of low temperature on human body and its physiological reactions are still researched. It is believed that exposure to cold significantly stimulates the hypothalamic-pituitary-adrenal (HPA) gland and sympathetic nervous system (SNS) activation and increases secretion of cortisol and catecholamines [46].

Wojtecka-Lukasik et al. confirm that the beneficial clinical effects of whole-body cryotherapy in patients with rheumatoid arthritis are in part due to the action on the processes of production, release, and the degradation of histamine [47].

Therefore, cold stress could modify levels of cytokines and immune responses, because cytokines play a crucial role in bidirectional communication between neuroendocrine and immune systems [48]. However, Straub et al. observed that these reactions proved to be insufficient in patients with rheumatoid arthritis [17].

In our research, RA patients demonstrated a significant decrease of IL-6 and TNF-*α* after a comprehensive treatment, regardless of the type of applied physical procedure. There are only several publications showing the effect of low temperatures on concentration of cytokines in patients with RA, and the results are inconclusive; Fricke et al. [49] and Lange et al. [50] observed a significant decrease of TNF-*α* level after WBC [17, 49]; and changes of IL-6 level were observed by Straub et al. [17] and Fricke et al. [49]. Interestingly, we observed, similar to Straub et al. [17], that IL-6 level measured after WBC in patients without glucocorticoids is significantly higher compared to those with glucocorticoids. This may result from abnormal HPA axes response in RA, and it is compliant with the results of Straub et al. [17]. Such effect was not observed in TNF-*α* level. It is worth mentioning that publications of other authors show no significant influence of kinesitherapy on immunological system [51].

Limitation of the study was small research group and some heterogeneity of this groups. This fact forces the authors to emphasize the need for caution in interpreting the presented results. Further studies are required to achieve confirmation of these findings.

## 5. Conclusion

The demonstrated results showed positive effects of a two-week rehabilitation program for patients with RA, regardless of the type of physical procedures. It is necessary to conduct studies on a larger number of people.

## Figures and Tables

**Table 1:** Baseline characteristics of the 44 patients with rheumatoid arthritis.

	Group I (*n* = 25): WBC group	Group II (*n* = 19): TR group	*p*
Age (years)	55.9 ± 5.08 (55; 51–60)	57.4 ± 5.3 (57; 52–61)	0.3373
BMI (kg/m^2^)	25.5 ± 2.71 (25.1; 24.1–26.8)	28.5 ± 4.78 (30.1; 23–32.9)	0.0565
DAS28	5.27 ± 1.13 (5.53; 4.15–6.18)	4.97 ± 0.87 (4.95; 4.22–5.58)	0.4409
Disease duration (years)	11 ± 5.37 (9; 8–15)	10.7 ± 9.2 (6; 3.5–18)	0.9058
RF positive, *n* (%)	17 (68)	13 (68)	
Treatment			
NSAID, *n* (%)	18 (74.1)	13 (68.4)	
DMARDs, *n* (%)	25 (100)	19 (100)	
Glucocorticoids, *n* (%)	16 (66.7)	16 (84.2)	
Prednisolone (mg/day)	4 ± 0.85	3.4 ± 0.94	

All data are expressed as mean ± SD (median; interquartile range) and percentages.

WBC: whole-body cryotherapy; TR: traditional rehabilitation; BMI: body mass index; DAS28: Disease Activity Score 28; RF: rheumatoid factor; DMARDs: disease-modifying antirheumatic drugs.

**Table 2:** Clinical characteristics of the two groups before and after the therapy.

		Group I WBC group			Group II TR group		Between groups
	Before	After	*p*	Before	After	*p*	*p*
Severity of pain (100 mm VAS)	51.64 ± 17.82 (53; 37–62)	40.80 ± 17.93 (37; 25–53)	0.0006^*∗∗*^	64.05 ± 18.36 (67; 49–80)	52.95 ± 16.36 (51; 40–62)	0.0003^*∗∗*^	0.7581
Duration of morning stiffness (min)	44.92 ± 47.35 (30; 3–60)	37.52 ± 47.41 (30; 3–30)	0.0157^*∗*^	81.32 ± 71.12 (60; 30–120)	58.68 ± 51.18 (40; 30–90)	0.0511	0.7312
HAQ-DI	1.82 ± 1.18 (1.88; 0.75–2.75)	1.64 ± 1.19 (1.63; 0.63–2.63)	0.0035^*∗∗*^	2.72 ± 1.48 (2.25; 1.5–3.75)	2.12 ± 1.30 (2; 1.38–2.75)	0.0029^*∗∗*^	0.0116^*∗*^
50-m test, walk time (s)	47.20 ± 11.39 (45; 40–55)	41.36 ± 8.67 (40; 35–45)	0.0001^*∗∗*^	56.11 ± 11.85 (50; 50–65)	51.79 ± 9.63 (50; 45–60)	0.0299^*∗*^	0.4205
50 m test, number of steps	80.32 ± 15.14 (76; 70–84)	76.20 ± 13.84 (73; 66–82)	0.0030^*∗∗*^	90.79 ± 9.62 (90; 84–99)	88.05 ± 10.08 (84; 82–96)	0.0457^*∗*^	0.6356
Severity of fatigue (100 mm VAS)	53.40 ± 18.95 (50; 45–62)	44.20 ± 21.76 (44; 25–60)	0.0034^*∗∗*^	63.11 ± 15.83 (62; 53–74)	48.47 ± 18.85 (42; 33–63)	0.0141^*∗*^	0.3871

All data are expressed as mean ± SD (median; inter quartile range). WBC: whole-body cryotherapy; TR: traditional rehabilitation; HAQ-DI: Health Assessment Questionnaire-Disability Index. ^*∗∗*^ *p* < 0.01, ^*∗*^ *p* < 0.05.

**Table 3:** Biochemical parameters in 2 groups of patients before and after the therapy.

		Group I WBC group			Group II TR group		Between groups
	Before	After	*p*	Before	After	*p*	*p*
ESR (mm/h)	25.52 ± 14.66 (25; 13–40)	20.96 ± 11.73 (20; 12–30)	0.0149^*∗*^	27.05 ± 13.66 (27; 17–35)	24.47 ± 12.87 (22; 17–30)	0.1416	0.3061
IL-6 (pg/mL)	17.96 ± 6.49 (18.59; 14.08–23.41)	11.75 ± 7.56 (13.60; 2.97–16.58)	0.0262^*∗*^	20.87 ± 5.43 (23.41; 15.95–24.92)	10.21 ± 7.92 (11.52; 2.73–13.04)	0.0076^*∗∗*^	0.1417
TNF-*α* (pg/mL)	11.77 ± 9.61 (11.29; 3.04–18.04)	4.46 ± 3.82 (3.84; 1.82–5.52)	0.0113^*∗*^	17.8 ± 19.54 (7.76; 3.84–24.45)	7.22 ± 14.74 (2.1; 1.33–4.19)	0.0258^*∗*^	0.8538

All data are expressed as mean (SD) (median; interquartile range). WBC: whole-body cryotherapy; TR: traditional rehabilitation; ESR: erythrocyte sedimentation ratio. ^*∗∗*^ *p* < 0.01,^*∗*^ *p* < 0.05.
